# Possible age-related differences in healthcare professionals’ perspectives on younger and older patients’ autonomy and decision-making in the context of sedation in specialised palliative care: exploratory secondary qualitative content and linguistic conversation analysis of interviews with healthcare professionals

**DOI:** 10.1186/s12904-022-00963-y

**Published:** 2022-05-13

**Authors:** Kurkowski Sandra, Heckel Maria, Pfaller Larissa, Peters Joachim, Bazata Jeremias, Schildmann Eva, Ostgathe Christoph

**Affiliations:** 1 Department of Palliative Medicine, CCC Erlangen – EMN, Universitätsklinikum Erlangen, Friedrich-Alexander-Universität Erlangen-Nürnberg (FAU), Erlangen, Germany; 2 Berlin, Germany; 3 Institute of Sociology, Friedrich-Alexander-Universität Erlangen-Nürnberg, Erlangen, Germany; 4 Chair of German Linguistics, Friedrich-Alexander-Universität Erlangen-Nürnberg, Erlangen, Germany; 5 Department of Palliative Medicine, University Hospital, LMU, Munich, Germany; 6 Charité – Universitätsmedizin Berlin, corporate member of Freie Universität Berlin and Humboldt-Universität zu Berlin, Department of Hematology, Oncology and Cancer Immunology, Oncological Palliative Care & Charité Comprehensive Cancer Center, Berlin, Germany

**Keywords:** End of life care, Palliative care, Age, Ageing, Autonomy, Qualitative content analysis, Linguistic conversation analysis

## Abstract

**Background:**

Chronic illnesses and multi-morbidity can threaten competence and independence, particularly in old age. Autonomy becomes increasingly important in the context of sedation, as in this case medication leads to (further) changes of consciousness. The study aimed to identify possible age-related differences in the perspectives of healthcare professionals on patients’ autonomy, in the context of sedation in specialised palliative care.

**Method:**

Secondary analysis of interviews with healthcare professionals, analysed by qualitative content and linguistic conversation analysis. The interviews analysed span 51 healthcare professionals in specialised palliative care across 17 centres (adult inpatient and specialist palliative home care services) in Germany.

**Results:**

The study shows that the perspectives of healthcare professionals on patients’ autonomy differs according to the age of the patient in the context of sedation in specialised palliative care. The different perspectives may lead to different ways of treating the patients, for example a greater space of autonomy and decision-making for younger patients.

**Conclusion:**

In particular, measures that may restrict consciousness (e.g. sedation) and thus influence patients’ ability to fully exercise their autonomy and fully participate in decision-making require special attention by healthcare professionals with respect to possible influences on treatment, such as different perceptions by healthcare professionals based on the patient’s age or age-related stereotypes.

**Trial registration:**

The study “SedPall” is registered in the German Clinical Trials Register (ID: DRKS00015047).

**Supplementary Information:**

The online version contains supplementary material available at 10.1186/s12904-022-00963-y.

## What is already known about the topic?

- The worldwide average life expectancy is continuously rising, leading to a growing proportion of older people in society. With this trend, possible age-related factors and special needs of older people become more important.
- In end-of-life care, communication, autonomy and decision-making are becoming particularly important. Multi-morbidity and/or need for care can restrict a person’s autonomy, especially when not compensated for by supporting environmental factors. This becomes even more important in the context of sedation as this can lead to changes in consciousness and consequently to restrictions patient’s expression of autonomy - for example in decision-making.
- In the context of the preferences and conditions for older people with co-morbidities to participate in medical decision-making the preferred role for older patients was for the doctor to make the final decision after considering the patient’s opinion (32.7%), and the most common actual role was that the patient was not asked for their opinion (27.5%). Thereby, the reason for the most common barrier for communication was the patient’s illness and not their age.

## What this paper adds

- In the study, two complementary qualitative analysis methods were combined (qualitative content analysis, linguistic conversation analysis) in an innovative and exploratory approach.
- The paper identifies age-related differences in perspectives on patients’ autonomy of healthcare professionals and the influence of those professionals on the autonomy and decision-making of younger and older patients in the context of sedation in specialised palliative care
- In addition to medical factors, it is important to sensitively consider other implicit factors that influence decisions and the involvement of patients in the decision-making process.

## Implications for practice, theory or policy

- To avoid age-discrimination it is important to encourage and support healthcare professionals’ self-reflection, and to raise awareness of it through training and education. Critical questioning of stereotypes can contribute to effective erosion of stereotypical patterns and instead to lead to the development of alternative mental structures.
- Intensified collaboration of geriatricians and gerontologists within palliative care may be instrumental
- In future, it has to be clarified where and how age discrimination comes into effect, and how it can be avoided. This requires further intersectional research.

## Background

Currently, the worldwide average life expectancy at birth lies at 73 years [1] and it is expected to rise, leading to a growing proportion of older people in society [2]. Hence e.g. in the healthcare sector possible age-related factors and special needs have to be addressed. With regard to the end of life, communication, autonomy and decision-making are becoming particularly important in the treatment and care of seriously ill people [3–5]. In situations at the end of life the question could arise as to which dimensions of autonomy must or should be promoted, preserved or protected. This is especially challenging when the ability to express one’s own autonomy, free will and free judgement is going to be limited [5]. This becomes even more important in the context of sedation as administering sedative medications can lead to or aggravate reduction of consciousness [6] and consequently to restrictions of communication, the ability to express one’s autonomy and to take part in decision-making.

Up until now, only two studies could be found on aspects of communication, autonomy and decision-making in the context of sedation at the end of life with a focus on age; however, neither refer specifically to specialised palliative care [3, 4]. De Gent et al. describe that “medical end-of-life decisions with possible or certain life-shortening effect [s]” [3] were “made for 53.6% very old (aged 80+) patients who died non-suddenly (vs. 63.3% for the younger patients)” [3]. These end-of life decisions “were not often discussed with the very old patients. Among competent patients this was less than compared with younger patients” [3]. Furthermore, data shows, that “[t] erminal sedation occurred among 6.9% of the cases, two times less frequently than for the younger patients” [3]. In a systematic review and meta-analysis Rietjens et al. concluded that “[a] mong patients older than eighty years, non-treatment decisions occurred more frequently compared with younger patients, while intensified symptom alleviation, palliative sedation, euthanasia/physician-assisted suicide and life-ending without explicit request were practiced less often” [4]. Additionally, the authors describe “that the administration of medication with a potential or certain life-shortening effect seemed generally to be practiced less often among the elderly, females and less well-educated patients compared with younger, male or more educated patients, while decisions that include the withdrawal or withholding of treatments seem to be more common in these groups” [4].

A Swedish study showed that the “most common preferred role [for the patient] was for the doctor to make the final decision after considering the patient’s opinion (32.7%), and the most common actual role was that the patient was not asked their opinion (27.5%)” [7]. Almost 40% of the older patients expressed that they would like to be given more information without having to actively ask for it, and 45% would have preferred to receive more information than they actually received during their last hospital stay [7]. In contrast, very few older patients did not want to receive any information about their medical treatment (3%) [7]. This study indicates that “the most common barrier to communication and thus with influence on participation in medical decision making was the patient’s own illness” [7], summarising that “preference for participation is highly individual, and age alone is no excuse for failing to invite the patient to participate in medical decision making” [7].

Mercandante et al. showed in an retrospective study an increase in opioid doses administered over the last days of the patient’s life in both age groups, but significantly more slowly in the age group of older patients (> 65 years) [8]. In total, 12.6% of this patient collective (*n* = 411) received (palliative) sedation to reduce consciousness. At this point, there was a significant difference between the age groups and more frequent (palliative) sedation among younger patients [8]. Symptoms for the indication were delirious disorders/confusion (82.7%) and respiratory distress (15.4%) [8]. The influencing factor of a younger age (mostly < 65 years) for a higher probability of (palliative) sedation at the end of life can be confirmed by further studies [3, 9–11].

With the aim “[t] o examine the presumed tension between care and concern for particular patients, and impartiality and equal concern for all patients also with regard to care for the elderly” [12] Skirbekk and Nortvedt conducted qualitative interviews with healthcare professionals in Norwegian hospitals and general practices [12]. They also discussed how “the professional thinking of medicine and nursing affects priority settings for the elderly” [12]. This study reveals that older patients not only have lower prioritisation in “potentially life-saving medical intervention [s]” [12] with regard to prioritisation decisions, but also a lower priority compared to younger patients in basic care [12]. These results indicate “that even if elderly patients were not openly discriminated because of their age, some important factors lead to different treatment for old and young patients” [12]. One explanation by the healthcare professionals is, that “elderly patients are not as likely to recover completely from their illnesses as younger patients. In general, complications are more probable, and elderly patients’ quality of life is not likely to improve as much from treatment as younger patients’ quality of life. Further, the risk associated with many forms of treatment becomes greater as the patients get older” [12]. Finally, taking into consideration “[h] ealth professionals’ assessments concerning quality of life, and patients’ and relatives’ preferences, cannot be devoid of value. Medical knowledge is not without implicit values and clinical judgment is always informed by norms. Informal and taken-for-granted norms for healthcare professionals’ decision-making might become problematic if alternative ways of prioritising are rarely considered or discussed. Ideals of care are generally associated with other-concern, empathy, contextual sensitivity, and competent moral perception” [12].

Especially in the context of the patients’ health condition and ability to assert autonomy and participation in decision-making becoming more limited, it seems really important to identify possible factors of influence for decision-making on the side of healthcare professionals, loved-ones and the patients’ legal representatives.

Therefore, this study aims to identify possible differences in the perspectives of healthcare professionals and their influence on the autonomy and decision-making of younger and older patients in the context of sedation in specialised palliative care. The study focuses on all forms of sedation in the context of specialised palliative care which are reducing the consciousness of the patients. A special category in the range of sedation is “continuous deep sedation”. “Palliative sedation” is defined by the European Association for Palliative Care as “the monitored use of medications intended to induce a state of decreased or absent awareness (unconsciousness) in order to relieve the burden of otherwise intractable suffering in a manner that is ethically acceptable to the patient, family and health-care providers” [13].

## Research design and methods

### Methodology

Answering the research question required an explorative design and the use of qualitative methods, since the constructs and concepts to be investigated are not yet sufficiently known in their combination and therefore a particular openness and flexibility towards the research object was required [14]. The interviews that form the basis for this analysis were conducted as part of the ‘SedPall’ consortium study in subproject two. With focus on the research question as well as openness and flexibility a first, random, review of the transcribed interviews took place by the first author of this study (SK, gerontologist). After reviewing the interviews for piloting and method reflection for the intended analysis, it became apparent that in addition to the explicit statements made by the healthcare professionals there were also implicit aspects, which could not be accounted for and evaluated by a qualitative content analysis. The theoretical interest of conversation linguists lies first of all in the reconstruction and explanation of conversational competence, but beyond that, conversation analysis also has the potential to be used in an application-oriented and practical way to remedy or improve for example conflicting or deficient communication processes [15]. Emotions determine a large part of the processes of perception, thoughts and actions and are, therefore, important in almost all areas of the human experience [16]. By means of language, emotions are expressed and named, aroused, intensified and constituted through specific representations [17]. Within linguistics, emotion linguistics therefore pertains to the question of how linguistic representations are used to infer inner emotional states and processes of human beings [17].

### Topic guide development and data collection

The qualitative interview topic guide was developed by two research associates (VH, PhD medical ethics, female / JB, MA sociology, male), with support from a senior researcher (ES, Medical doctor, MSc palliative care, female) and the multi-professional research consortium, based on available literature and the researchers’ working experience. Both VH and JB were trained and experienced in the development of topic guides as well as conducting and analysing interviews. Qualitative researchers in the department and a qualitative expert group at LMU university were also consulted for feedback and to facilitate self-reflection. The final topic guide (see as example for physicians supplement material 1) covered five main topics, with some variance regarding different professions: Experience with sedation and participant’s understanding of sedation in specialist palliative care, indications for and intentions behind sedation therapy, decision-making process and consent, challenges and opportunities, dying under sedation. The topic guide was intended to create an environment for the participants to describe their experiences with and views on sedation as openly as possible. The topic guide was piloted in six interviews, with VH, JB and ES revising the guide where appropriate.

Recruitment took place in 10 palliative care units and seven specialist palliative homecare teams in 12 German cities. In each participating centre, a local contact person (often an experienced MD or nurse who knew the team well) was named and involved in identifying and establishing contact with potential interview candidates. Potential candidates were contacted by either JB or VH, informed of the study’s aims, the interviewer’s experience and credentials and the interview setting. The participants had the opportunity to ask questions regarding the study and gave their oral and written informed consent prior to the interview. The inclusion criteria were experience with at least one case of sedation and sufficient command of the German language. Purposeful sampling was implemented to ensure a varied sample regarding profession, position, age, gender and working experience.

The interviews were conducted by VH and JB face-to-face in the participants’ workplaces between July 2018 and September 2019, often in blocks of 2–4 interviews per recruitment centre. During the interview, no other personnel was present. Field notes were written and compared after each interview or block, in which personal observations and possible reactions or biases were also included. Four scheduled interviews had to be cancelled because of illness or unexpected scheduling conflicts. The interviews’ duration ranged from 45 to 90 minutes. No repeat interviews were conducted. During the interview process, the researchers engaged in constant self-reflection and discussed whether new and important themes had emerged that made changes to the topic guide necessary and whether data saturation had been achieved, which constituted the end of the interview period.

All interviews were audio-recorded and transcribed verbatim, including anonymisation. Data on sociodemographic and professional background were collected using an anonymised questionnaire. The participants did not receive the anonymised transcripts. Ethical approval was given by local ethics committee (reference number 18–191,19.04.2018) and the study was performed in accordance with the relevant guidelines and regulations (Declaration of Helsinki). The COREQ checklist was followed to ensure methodological rigour (see supplement material 2).

### Participants

The database for the secondary analysis is 51 interviews on sedation with healthcare professionals in the field of specialised palliative care (adult inpatient and specialist palliative home care services) in Germany. Interviewees (*n* = 51) were between 25 and 64 years old (on average: 48 years) and mainly female (*n* = 33 / male *n* = 18). They worked in specialised inpatient palliative care (*n* = 32), specialised palliative home care services (*n* = 18) or both settings (*n* = 1). They were physicians (*n* = 23), nurses (*n* = 20) or other professionals (in total *n* = 8. Physiotherapists *n* = 2; psychologists *n* = 2; respiratory therapists *n* = 1; spiritual caregivers *n* = 3). Their working experience in general ranges between 2 and 40 years and lies on average in 21 years. In the field of palliative care their working experience ranges between 0,75 and 23 years and lies on average in 8 years.

In order to analyse explicit expressions within the interviews regarding aspects of age- related differences in perspectives of healthcare professionals on patients’ autonomy, a secondary qualitative content analysis [18] with an constructivist approach was used. To go beyond and systematically reveal even implicit notions between the lines, the content analysis was complemented by a linguistic conversation analysis focusing on linguistic representations of age-related differences in perspectives of healthcare professionals on patients’ autonomy. Since this secondary analysis was not planned during the initial topic guide development, the data were not collected with a specifically gerontological focus and therefore, they were used for secondary analysis in this work. As a result of the explorative characteristics of the research at hand, two qualitative research methods (qualitative content analysis, linguistic conversation analysis) were combined to achieve a complementary viewpoint [19].

### Qualitative content analysis

**Fig. 1:**
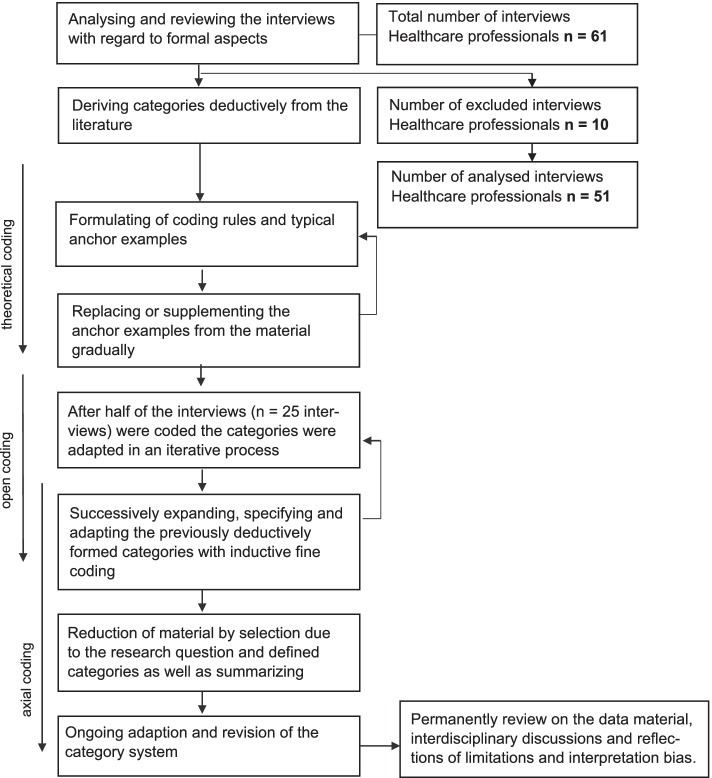
Process of data analysis. Qualitative content analysis

Following the process model of structuring qualitative content analysis (see Fig. 1), at the beginning all interviews (*n* = 61) were analysed with regard to formal aspects [18]. These were sociodemographic information and references to the chronological age of the interviewees in the context of field notes. After this step 10 interviews had to be excluded because there were no references from the healthcare professionals to the age of the reported patients (*n* = 10). Thereafter, 51 Interviews remained which could be analysed. This was followed by an assessment of the key topics in relation to the research question (autonomy, decision-making) within the interviews. As the interviews were not originally conducted with these key topics, this step was important to allow further analysis. On reviewing the interviews, the age groups were derive from literature [20–22] determined as: patients with a chronological age of less than 60 years were coded as ‘younger patients’, while patients with a chronological age of greater/equal 60 years were coded as ‘older patients’. Patients were also coded according to the respective attributions of the healthcare professionals (e.g. ‘younger patient’ into the group ‘younger patient’). For the coding process MAXQDA software (version 20) [23] was used by one researcher (SK) in close discussion and collaboration with the other authors of the study. Following the process model, categories were deductively derived and defined from previous theoretical knowledge and the state of the literature, and coding rules were formulated. In addition, typical anchor examples were formulated in advance in the main categories. In the course of the coding process, these were gradually replaced or supplemented by anchor examples from the material. After half of the coded material (25 interviews), the previously deductively-formed categories were thus successively expanded, specified and adapted within the framework of inductive fine coding. In this way, the iterative process necessary for the coding process became an interplay of fine coding and specification with the material. The coding method was initially strongly theoretically coding and then became increasingly open and axial [19].

### Linguistic conversation analysis

**Fig. 2:**
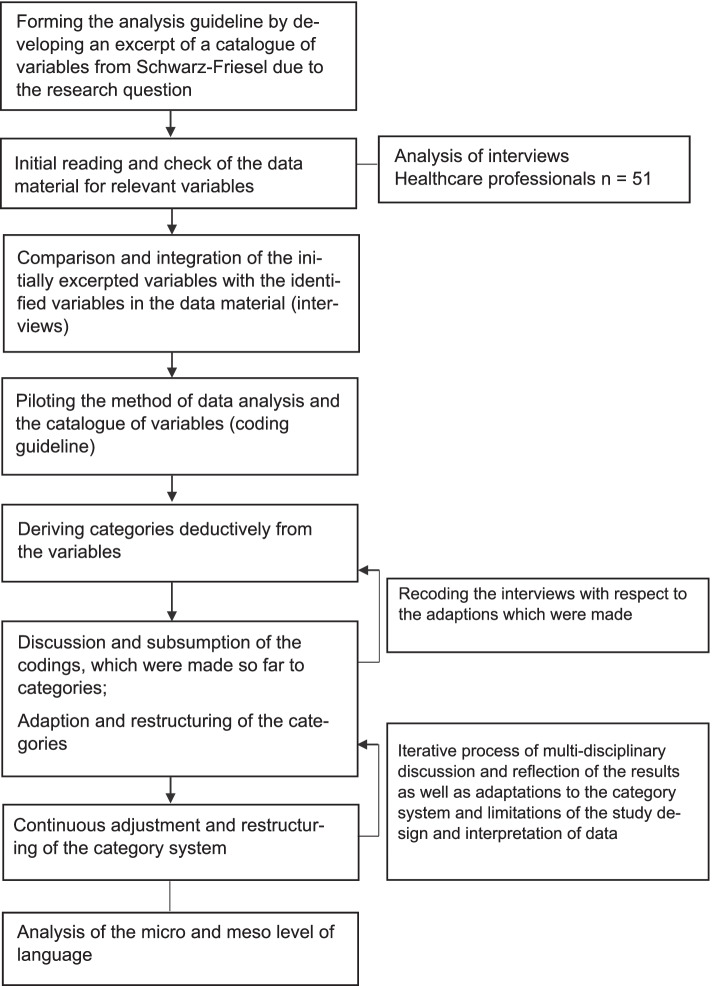
Process of data analysis. Linguistic conversation analysis

The parts of the interviews with a reference to age were additionally linguistically analysed using MAXQDA (version 20) [23]. A the first step, the analysis guideline was formed by developing an excerpt of a catalogue of variables from the emotion linguist Schwarz-Friesel [24]. The guiding principle was thereby the research question. This step was followed by an initial reading where the material was checked for relevant variables [25]. The identified relevant variables were then compared with the excerpt prepared according to Schwarz-Friesel [24] and integrated with each other. In the next step, categories were deductively derived from these variables for the analysis. Subsequently, the regular coding process started. The coding process focused on the micro and meso level of language (micro level: individual words, meso level: sentences and paragraphs). In analysing the interviews, the macro and para textual level were not considered, as these were presented in the context of the material at hand (transcribed interviews) to a degree that was not relevant to the research question during the initial reading. A detailed process model of linguistic conversation analysis is attached (see Fig. 2). The entire coding process was monitored by a linguistic researcher acting in an advisory capacity and, for quality assurance purposes, reflected upon, discussed and the whole research process documented as well as in its individual steps. The criteria of rule-guidance and intersubjective comprehensibility were also central in this analysis process. In order to enable rule-guided and intersubjective comprehensibility, a detailed research diary (logbook) was also kept with a note of the steps taken in each case and any necessary justification. Additionally, in this logbook notes of conversations, discussions, agreements as well as necessary changes, decisions and adjustments were recorded. The coding trees for qualitative content analysis and linguistic conversation analysis are not provided in this manuscript.

## Results

### Qualitative content analysis

Regarding possible age-related differences in healthcare professionals’ perspectives on patients’ autonomy a difference can be seen between younger and older patients. While patient autonomy is emphasised in cases of younger patients, and even advocated for –in this case by the specialised palliative care physician towards the primary care physician– the data set shows a tendency of devaluation of older patients’ autonomy, consequently restricting their range of decision-making and action. Thus, for younger patients:

‘*[T] he primary care physician stated: ‚I won’t draw any blood from him, he’s palliative anyway and besides, what’s up with these transfusions?‘ So I said: ‘This man is of clear mind, born in ‘72, yes, he’ll tell you exactly what he does and doesn’t want.’ ( … ) And that’s what the rest of the procedure is based on, it doesn’t work any other way”* (P1).

On the other hand, for older patients, the following example shows attempts to minimise the patient’s expressed symptoms. The patient’s insistence is viewed negatively, until in this case, a doctor decides to refuse the patient’s treatment – in a more paternalistic than advocatory way:

*“[H] e always wanted to be sedated, wanted to get the injection, so that he could fall asleep and not wake up, was 80 years old. (...) It was only the loss of autonomy, in the end, that was left as an indication, because he didn’t have much pain or nausea. So really he didn’t have anything, aside from, yeah, not being able to deal with the situation. And it was exhausting, every day, because every day he wanted to have the lethal injection, and it was very difficult to get him away from that. But then I decided not to sedate him”* (P2).

Beyond that, the description of the stress experienced in attending to an older patient and the perceived emotional burden seems to be lower, where on the other hand the attending to a younger patient is described as emotionally more burdensome, as it is illustrated in the following expressions:

*“[W] hen it’s just end-of-life care, when they’re 95, where everyone can, I think, follow along relatively well. And you don’t take it home with you”* (P4).

*“And I think, others in palliative medicine will see it the same way, particularly when you have a young person in front of you, you have a pit in your stomach and the decision [sedation], leaves an impression before and long after”* (P1).

This difference in perception and evaluation of patient autonomy between younger and older patients by healthcare professionals was also seen on a semantic level.

### Linguistic conversation analysis

Regarding the attribution of autonomy, it is important to take the semantic roles into consideration that healthcare professionals ascribe to younger as well as older patients. Although sedation is a medical treatment that cannot be done by the patients themselves actively, the active and passive formulations of sentences show a different degree of involvement of the patients. Thereby, younger patients are assigned an active role in sentence structures and are given an acting role. For example, a healthcare professional describes in the case of a younger patient:

*“And then she still wanted to, because she already knew, if she gets sedated now, that she wouldn’t see her parents again. And then we just had one more week for them to really have a proper farewell, to take all of it in, even with all the downsides, but then we accept that, because it’s just how they want it”* (P5), *“then in the end we offered to bring her to the ward with us. That’s where she was finally palliatively sedated”* (P7) or *“in fact, we agreed on it together, that we would sedate her, yes”* (P8).

In contrast, the tendency for older patients in the sense of a speech act analysis is to put them in a passive role with a lower degree of involvement: *“Now, in the other case, with Mr. [name], he was already diapered”* (P3) or phrases in connection with pressure, force, or manipulation such as: *“[W] e got it so far, that she (...) accepted the syringe driver”* (P1). In other cases, the passive role of the patient was also applied to relatives: *“[B] ut then the carers or relatives are told: ‚Now we would sedate.”* (P5) or *“they will get the patient ready and sedated from us”* (P6).

This shift of communication from patient to relatives can also happen in younger patients’ cases, but without the pejorative use of language commonly used with older patients and the resulting constraints on their autonomy. In connection with pressure or force, the chosen phrases for younger patients are more lenient or attenuated, such as:

*“But it was very clear that she didn’t want that at all and (...) then we initially just discontinued use of pain medication at home”* (P9), *“and then (...) we considered a palliative sedation”* (P10) or *“that she was then brought into a sedation”* (P11).

In accordance with younger patients’ autonomy, passages with active or reported speech can be found in this context: *„He just said: ‚I gotta go, I need some deep sleep, I can’t take it anymore, (...) “*(P6) or *„he even said that he wanted, he wanted to be sedated “*(P12), whereby patients can speak for themselves and are somewhat literally quoted. There are also differences in the choice of how reassurance is phrased between the age groups. For younger patients, the tendency is towards choosing phrases carefully while at the same time weakening the negative connotations of them: *„[H] ow shall I say? “*(P8), *„to exaggerate a bit “*(P12) or *„this is kind of a stupid way of putting it, but (...) “*(P13). In contrast, the tendency in phrases for older patients is more absolute in nature with a more justifying claim, such as: *„to be honest “*(P5), „*I have to say* “(P14) or „*as one may say* “(P15).

In addition to the results of the qualitative content analysis, the linguistic conversation analysis also shows the differences in the perceived emotional burden in attending to younger and older patients. Healthcare professionals are found to use normalising expressions in relation to the dying process of older patients, in contrast to the emphasised tragedy and burden of the dying process of younger patients, or in their contrasting youth with frailty, such as:

*“[S] he was such a shrivelled old granny “*(P5) versus *“Even just seeing that, I mean, he was in his mid-thirties and to see himself in the mirror, disfigured by his tracheostomy tube, (...). Well that’s pretty tough “*(P12).

Aside from an uneven distribution of semantic roles – demonstrating a tendency of passive roles for older patients the interviews showed differences in the perspectives of healthcare professionals of older patients and younger patients.

## Discussion

To the best of the authors’ knowledge, this is the first study that focuses on possible age-related differences in healthcare professionals’ perspectives on younger and older patients’ autonomy and decision-making in the context of sedation in specialised palliative care. For this purpose, an innovative study design was developed in multi-professional and interdisciplinary collaboration, combining various qualitative evaluation methods.

The results of the qualitative data analysis shows that healthcare professionals describe more deficit-oriented perspectives of older patients, whereas a focus on resources and aspects of vitality and youthfulness was found toward younger patients. Also a report published by Denninger describes that with older patients, aspects of dependency, need for help and care are particularly emphasised, while with younger patients, their desired or actual independence is emphasised [26]. Ideas of normality are central to the construction of categories of equality and inequality [26]. Hierarchies and structures of power are always inscribed in these, for example, normative ideas of a ‘normal’ body or a ‘normal’ life [26]. For ‘old age’, analogous to the category ‘disability’, strong tendencies towards protonormalism (“orientation towards inflexible and fixed norms”, [26]) can be found. Impairments in old age seem to violate an implicit norm to a lesser extent and are therefore perceived as less severe [26]. Partly because of this, in our results healthcare professionals seem to be less burdened by the consultation of, treatment and sedation of older patients. Lux et al. summarise that decisions of a therapy-limiting nature seem to be easier for older people than for younger patients, since the end of life is more likely to be perceived as natural and also inevitable [27].

In relation to autonomy, the results of the analyses show that there are age-related differences in the perspectives of healthcare professionals on patients’ autonomy. For example, healthcare professionals tended to perceive demanding, loud and strong-willed behaviour in younger patients as rather positive in the sense of ‘standing up for oneself and one’s loved-ones’, whereas in older patients this is perceived as rather negatively and is classified as rebellious. These results are consistent with the findings of Mayer and Rothermund, according to whom observed behaviour can be explained by activated age stereotypes and their prescriptive character, but can also lead to misattributions [28]. This could explain, why older patients in the interviews seemed to be less involved in aspects decision-making. Further supporting this finding, de Gendt et al. report, that compared to competent younger patients, competent very old patients (aged 80 years or older) were less frequently involved in end-of-life decision-making (56.1 vs. 71.9%, *p* = 0.017) [3]. Medical end-of-life decisions with a possible or certain life-shortening effect among the oldest age group were less frequently discussed with another physician than in younger patients (34.9% vs. 53.9%, *p* = 0.003) [3].

With regard to an over-adaptation and a protective, paternalistic attitude, which is, however, based on a deficit conception of ‘old age’, this can be named as a form of positive ageism, as this also undermines the independence and self-worth of older people in the long run through overprotective behaviour [28]. Kitwood formulated both the tendencies of over-adaptation in communication as well as in paternalistic behaviour [29]. In his 17 points of malignant social psychology, he included forms of over-adaptation, such as not allowing people to use their remaining abilities and skills or withholding information from them [29]. This can be seen in the over-adaptations that have become visible in the interviews in form of a lower degree of involvement of older patients in questions of decision-making as well as less severely regarded symptoms in older patients.

Another reason for the rather early informing of younger patients about the possibilities of (palliative) sedation, instead of their age and thus a bias, could be due to the presence of certain diseases, such as COPD (Chronic Obstructive Pulmonary Disease) or ALS (Amyotrophic Lateral Sclerosis). These diseases are described as particularly uncertain and difficult to predict with regard to possible courses and symptom exacerbations [30, 31]. This is accompanied by patients who are already well informed about the chronic condition and therefore the ongoing course of the disease with regard to various treatment and therapy options [30–32].

Almost regardless of age, the interviews reveal challenges with psycho-socially related symptoms for the indication of (palliative) sedation. Overall, the literature reveals a controversial discussion on the implementation of palliative sedation in cases of predominantly to exclusively present psychosocial symptom burden [33–35]. Challenges that further complicate decision-making for palliative sedation in this context are described by Bruce & Boston as the uncertainties in the definition of ‘existential suffering’, the highly subjective expressions of it, and the different personal experiences of physicians and nurses with regard to (experienced) despair and vulnerability [34].

Due to the explorative character of this study as well as the study design of secondary analysis the results should be confirmed by further research, with particular focus on the connection between stereotypes and images of aging. Furthermore, it has to be clarified where and how exactly age discrimination comes into effect, and how, in the future, it can be avoided.

### Strengths and limitations

An essential strength of this study is the innovative approach of combining the methods of qualitative content analysis and linguistic conversation analysis. In the case of sedation of older patients, it is evident that the description of decision-making processes corresponds to the semantic level. Another strength can be found in the large number of interviews with healthcare professionals in specialised palliative care which were analysed for this study. In addition to this, not having a specifically gerontological focus during the interviews uncovers in which contexts the calendar age of the patients is taken into consideration and becomes meaningful for the healthcare professionals. To analyse and contextualise the results, it is important to disclose the epistemological positions of the main researchers involved. On the part of the doctoral candidate (first author), these were from a background of social science (social work) as well as from gerontology and palliative care. The backgrounds of the other researchers intensively involved were: nursing science, sociology, medicine and linguistics. Through this interdisciplinary exchange, it was possible to discuss the results and their interpretative uncertainties throughout the entire research process.

On the other hand, this project faces the limitation of selection bias, as it was easier for interviewees to remember younger patients. This led to the availability of more data on younger patients for this secondary analysis. In addition, using interviews with remembered cases the likelihood of examples or cases responding to stereotypes is greater than in observations. Using secondary analysis also precludes asking further questions and exploring respondents’ statements from the perspective of the research question. In this study it would have been necessary to ask questions about the intentions and influencing factors behind the statements of the healthcare professionals. Thus, this study can only speak of tendencies and no causal connections can be made. Another limitation is the age limit of 60 years, which was set and derived from literature as well as the respective attributions of the healthcare professionals in age groups (e.g. ‘younger patient’ into the group ‘younger patient’). This has to be further investigated in detail in another study design (primary study design) with a special focus on gerontological issues, differences and concepts. Also, the average of age of the healthcare professionals seems to be rather high at 48 years. This also could point to a selection bias. Looking at the generalisability of the results, there are limitations due to the care context being specialised palliative care and the small number of interviewees within specialised groups of healthcare professionals. Specific limitations in the linguistic analysis are due to the fact that aspects of emotionality in the language were considered and analysed, however without being able to connect these results with the underlying intention of the statements. Therefore, the results of this study are to be regarded cautiously and should be examined and confirmed by further in-depth research, with particular focus on the connection between stereotypes, self-images, and portrayals of aging.

## Conclusion

The results of this study indicate that healthcare professionals have different perceptions of younger and older patients’ autonomy. This becomes important in the context of sedation as administering sedative medications can lead to (further) restrictions of consciousness and consequently to restrictions of communication, the ability to express one’s autonomy and to take part in decision-making. Therefore, in addition to medical factors, it is important to sensitively consider other implicit factors that influence decisions and the involvement of patients in the process of decision-making. It is possible that a permanent critical questioning of stereotypes can contribute to an effective erosion of stereotypical patterns and instead to lead to the development of alternative mental structures. Furthermore, it has to be clarified where and how exactly age discrimination comes into effect, and how, in the future, it can be avoided and healthcare professionals can be supported. This requires further, and particularly, intersectional research.

## Supplementary Information

**Additional file 1.**

**Additional file 2.**

## Data Availability

The datasets generated and/or analysed during the current study are not publicly avail-able due to the fact that the doctoral thesis has not yet been completed, but are available from the corresponding author Sandra Kurkowski via E-Mail (sandrakurkowski@web.de) on reasonable re-quest.
