# Correction: Possible age-related differences in healthcare professionals’ perspectives on younger and older patients’ autonomy and decision-making in the context of sedation in specialised palliative care: exploratory secondary qualitative content and linguistic conversation analysis of interviews with healthcare professionals

**DOI:** 10.1186/s12904-022-00990-9

**Published:** 2022-06-01

**Authors:** Sandra Kurkowski, Maria Heckel, Larissa Pfaller, Joachim Peters, Jeremias Bazata, Eva Schildmann, Christoph Ostgathe

**Affiliations:** 1 Department of Palliative Medicine, CCC Erlangen – EMN, Universitätsklinikum Erlangen, Friedrich-Alexander-Universität Erlangen-Nürnberg (FAU), Erlangen, Germany; 2 Berlin, Germany; 3 Institute of Sociology, Friedrich-Alexander-Universität Erlangen-Nürnberg, Erlangen, Germany; 4 Chair of German Linguistics, Friedrich-Alexander-Universität Erlangen-Nürnberg, Erlangen, Germany; 5 Department of Palliative Medicine, University Hospital, LMU, Munich, Germany; 6 Charité – Universitätsmedizin Berlin, corporate member of Freie Universität Berlin and Humboldt-Universität zu Berlin, Department of Hematology, Oncology and Cancer Immunology, Oncological Palliative Care & Charité Comprehensive Cancer Center, Berlin, Germany

Correction: BMC Palliative Care 21, 1 (2022).

10.1186/s12904-022-00963-y

Following the publication of the original article [1], the author reported that the given name and family name of all authors were erroneously transposed.

The author group has been updated above and the original article [1] has been corrected.
