# Supplementary material for: Possible age-related differences in healthcare professionals’ perspectives on younger and older patients’ autonomy and decision-making in the context of sedation in specialised palliative care: exploratory secondary qualitative content and linguistic conversation analysis of interviews with healthcare professionals
Source: BMC Palliat Care. 2022 May 13;21:71. doi: 10.1186/s12904-022-00963-y (PMC9101857; doi:10.1186/s12904-022-00963-y)
Supplement: Supplementary file 1 — Additional file 1. [file 12904_2022_963_MOESM1_ESM.pdf]

SedPall – Interview guide for healthcare professionals: physicians*

Key questions (narrative prompt)	Check - Memo for possible follow-up questions
**Part 1: Experiences with sedation in specialist palliative care and understanding of the concept**	
What experience have you gained with sedation in your work environment?	Experiences<br>Descriptions<br>Emotions, own experiences<br>Protocol/Guidelines
Can you describe in your own words what distinguishes sedation in palliative care for you?	Definition/Understanding<br>Different types of sedation/spectrum of sedation measures<br>Terms such as palliative sedation, terminal sedation, continuous/intermittent
**Part 2: Indication**	
When do you usually sedate a patient?	Conditions<br>Time in the course of the disease<br>Intention/aim<br>Restraint<br>Patient's request<br>Refractory symptom<br>(Unbearable) suffering<br>▪ Subjective vs. objective suffering<br>▪ Difference between physical and psycho-existential symptoms as the cause of suffering<br>Demarcation: unjustified sedation
**Part 3: Decision-making and consent**	
Based on your experience, how is the decision made to sedate a patient?	Allocation of roles in decision-making<br>Disagreement/conflict situations<br>Time pressure during decision-making due to a high burden of symptoms?<br>Weighing the benefits/risks<br>Weighting awareness<br>Life-shortening effect
If you have informed the patient and, if applicable, their relatives in the past that sedation is being considered, how did you do this?	Informed consent of patient and, if applicable, relatives<br>Health care proxy<br>Judicial guardian<br>Contents of the informed consent discussion<br>Autonomy in the case of unbearable suffering

**Part 4: Challenges and opportunities**<br><br>What have you found particularly difficult or challenging in your experience of sedation?<br><br>What have you found to be particularly positive about sedation cases?	Nutrition and hydration<br>Checking the outcome<br>Letting the patient wake up<br>Monitoring<br>Effect on treatment team<br>Demarcation from euthanasia
**Part 5: Contextual aspects**<br><br>In your experience, do patients with and during sedation die differently from other patients in your daily practice?	Demarcation from "natural death"<br>Procedures/Rituals/Farewells<br>Perception of the patient
**Part 6: Conclusion/Reflection**<br><br>When you look back on our conversation: What was a particularly interesting or important aspect for you? Why?<br><br>Is there anything else you would like to say about this subject that we have not addressed?	Reflection on conversation

*Interview guides were developed for nurses, physicians, and other members of the multiprofessional care team, with slight adaptions, respectively.
